# Spiders do not escape reproductive manipulations by *Wolbachia*

**DOI:** 10.1186/1471-2148-11-15

**Published:** 2011-01-14

**Authors:** Bram Vanthournout, Janne Swaegers, Frederik Hendrickx

**Affiliations:** 1 Terrestrial Ecology Unit, Department of Biology, Ghent University, Ledeganckstraat 35, 9000 Ghent, Belgium; 2 Royal Belgian Institute of Natural Sciences, Vautierstraat 29, 1000 Brussels, Belgium

## Abstract

**Background:**

Maternally inherited bacteria that reside obligatorily or facultatively in arthropods can increase their prevalence in the population by altering their hosts' reproduction. Such reproductive manipulations have been reported from the major arthropod groups such as insects (in particular hymenopterans, butterflies, dipterans and beetles), crustaceans (isopods) and mites. Despite the observation that endosymbiont bacteria are frequently encountered in spiders and that the sex ratio of particular spider species is strongly female biased, a direct relationship between bacterial infection and sex ratio variation has not yet been demonstrated for this arthropod order.

**Results:**

Females of the dwarf spider *Oedothorax gibbosus *exhibit considerable variation in the sex ratio of their clutches and were infected with at least three different endosymbiont bacteria capable of altering host reproduction i.e. *Wolbachia*, *Rickettsia *and *Cardinium*. Breeding experiments show that sex ratio variation in this species is primarily maternally inherited and that removal of the bacteria by antibiotics restores an unbiased sex ratio. Moreover, clutches of females infected with *Wolbachia *were significantly female biased while uninfected females showed an even sex ratio. As female biased clutches were of significantly smaller size compared to non-distorted clutches, killing of male embryos appears to be the most likely manipulative effect.

**Conclusions:**

This represents to our knowledge the first direct evidence that endosymbiont bacteria, and in particular *Wolbachia*, might induce sex ratio variation in spiders. These findings are pivotal to further understand the diversity of reproductive phenotypes observed in this arthropod order.

## Background

Maternally inherited endosymbiont bacteria of arthropods received considerable attention owing to their ability to shape their hosts' reproductive biology and consequently their ecology and evolution [1-4]. The strategies adopted by these microorganisms to increase their fitness are surprisingly diverse and involve the induction of parthenogenesis, killing of male offspring, feminization of genetic males and cytoplasmic incompatibility, wherein, in its simplest form, the development of an uninfected egg is inhibited if inseminated by sperm of an infected male [2,4,5].

Recently, several molecular screening studies demonstrated that the degree of arthropod infection is considerably higher than previously thought [6,7] and that even up to 66% of arthropod species are thought to be infected [8]. Despite the discovery of their widespread occurrence within arthropods, knowledge about the extent to which they may alter their hosts' reproduction is lagging behind. Even for some large taxonomic groups, such as the order of spiders (Araneae), conclusive evidence is at present lacking. Nevertheless, some spider species exhibit reproductive phenotypes similar to those expected under endosymbiont infection such as parthenogenesis and primary sex ratio distortion in social as well as solitary species (e.g. [9-15] and see [16] for an extensive overview). In particular for solitary species, primary sex ratio distortion is commonly expected to result from reproductive manipulation by endosymbionts as Fisher's sex-allocation theory generally predicts that an equal sex ratio is the only evolutionary stable outcome from the host's perspective. Moreover, the prevalence and diversity of endosymbiont bacteria in spiders is among the highest within the arthropods and up to five different endosymbionts capable of manipulating their hosts' reproductive biology have been found in several spider families: *Wolbachia, Rickettsia, Cardinium, Arsenophonus *and *Spiroplasma *[6,16-20].

Causal relationships between endosymbiont infection and sex ratio distortion in spiders are up till now only suggested by a difference in their prevalence between males and females [6] or by an indirect relationship [21]. Yet, as many other factors beside endosymbionts might cause sex ratio distortion [1], multiple lines of evidence such as maternal inheritance of sex ratio variation, use of different antibiotics that target an array of different bacterial families and a direct relationship between endosymbiont presence and sex ratio effect are necessary to disentangle the impact of each endosymbiont on the produced sex ratio [5,22].

In this study, we report on sex ratio variation in the solitary spider *Oedothorax gibbosus *(Araneae: Linyphiidae: Erigoninae). This small dwarf spider has a palearctic distribution and occurs exclusively in damp habitats such as marshes and wet forests, where they reside in grass tussocks and patches of moss situated close to the water. Besides the observation that the species exhibits a clear male dimorphism with alternative mating strategies [23], previous research showed primary sex ratio distortion with an excess of females [24]. Here, we explore the potential role of endosymbionts in inducing this sex ratio variation by (i) unraveling the inheritance pattern of the sex ratio trait, (ii) relating the presence of several endosymbiont bacteria with sex ratio variation and (iii) investigating whether an equal sex ratio can be restored by antibiotic treatments.

## Results

### (i) Maternal inheritance of sex ratio variation

Previous studies showed that sex ratio in *O. gibbosus *is significantly female distorted, but varies considerably among clutches [24]. If this observed sex ratio distortion is caused by maternally inherited endosymbiont bacteria, only daughters should inherit the sex ratio trait.

By means of an animal model (see Methods section) applied to our extensive pedigree data, the variance in sex ratio among females that is not attributed to sampling error is decomposed into a maternally inherited part ($\sigma_{m}^{2}$), which only incorporates this part of the sex ratio variation that is transmitted to their daughters, and a residual part ($\sigma_{e}^{2}$) that estimates the remaining variation. Possible causes of $\sigma_{e}^{2}$ to deviate from zero include non-random variation in sex ratio among dams, for instance due to loss of the bacterium, genetic variation in resistance, direct paternal effects or other causes of sex ratio variation.

**Figure 1:**
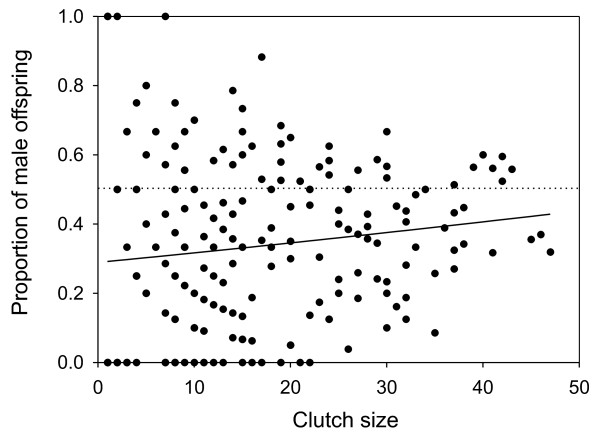
**Relationship between proportion of male offspring in a clutch and size of the clutch in the dwarf spider *Oedothorax gibbosus***. Solid line depicts the linear correlation. The dotted line depicts a 50:50 sex ratio and is given for illustrative purposes.

The average sex ratio equalled 0.34 (95% CI: 0.31 - 0.36) and is hence significantly lower than 0.5, but showed considerable variation among clutches (Figure 1). Posterior densities of both variance components show that the largest source of sex ratio variation consists of the maternally inherited part. As this variance is much higher than zero ($\sigma_{m}^{2}$; mean: 0.64; 95% CI: 0.34 - 1.05), this is evidence that the observed sex ratio variation in this species is strongly maternally inherited. However, a smaller but still highly significant residual sex ratio variation ($\sigma_{e}^{2}$) was observed (mean: 0.27; 95% CI: 0.12 - 0.47), indicating that not all sex ratio variation in this species is maternally inherited and that other factors of minor magnitude are additionally responsible for the among female variation.

The clutch sex ratio was significantly related with the size of the clutch (weighted Pearson correlation; *r *= 0.18; *P *= 0.01), with the proportion of sons being significantly lower in smaller clutches (Figure 1).

### (ii) Endosymbiont identification and prevalence

Single individuals, sampled at two independent populations, tested positive for up to three different endosymbionts capable of altering host reproduction i.e. *Wolbachia*, *Cardinium *and *Rickettsia*. For *Wolbachia*, both sets of primers (i.e. *wsp *and *Wolbachia *specific 16S ribosomal DNA) gave consistent results. Sequences of both genes could be read unambiguously and no among individual variation was observed within the obtained *wsp *and *Wolbachia *specific 16S sequences ([GenBank:HQ286290] and [GenBank:HQ286291] respectively). This suggests that a single *Wolbachia *strain is present in infected individuals. BLAST searches for both genes returned the highest matches with available *Wolbachia *sequences (E-values < 1e-199). For *wsp*, the obtained sequence clustered within supergroup G (See additional file 1: Bayesian inference tree of *Wolbachia *wsp sequences), which according to Rowley et al [19] primarily comprises *Wolbachia *endosymbionts of spiders. The recognition of this supergroup as a monophyletic clade has however recently been debated [25]. Sequences were most closely related with *wsp *sequences found in the spiders *Diaea circumlita *[GenBank:AY486092] and *Hylyphantes graminicola *[GenBank:EU723842], the nematode *Angiostrongylus cantonensis *[GenBank:AY508980] and the mosquito *Malaya genurostris *[GenBank:AY462865] (See additional file 1: Bayesian inference tree of *Wolbachia *wsp sequences). For 16S, the sequence could not be classified unambiguously into one of the supergroups as defined in Lo *et al *[26] and was most closely related to the *Wolbachia *16S sequence found in the spider *Tetragnatha montana *[GenBank:EU333940](See additional file 2: Bayesian inference tree of *Wolbachia *16S sequences). Similarly, sequences for *Rickettsia *and *Cardinium *were easily readable and showed no variation ([GenBank:HQ286289] and [GenBank:HQ286292] respectively). The *citrate *partial sequence of *Rickettsia *clustered within a monophyletic group that consists almost exclusively of *Rickettsia *sequences obtained from other spiders (See additional file 3: Bayesian inference tree of partial *citrate *sequences of *Rickettsia*). The phylogenetic position of the *Cardinium *sequence could not be positioned with high support among one of the other endosymbiont *Cardinium *sequences available at GenBank (See additional file 4: Bayesian inference tree of *Cardinium *16S sequences). For *Spiroplasma*, only a few faint bands were visible after electrophoresis. However, sequencing and BLAST searches revealed that these were false positives and due to amplification of the bacteria *Acidovorax*.

Approximately half of the individuals were infected with *Wolbachia *with 44% (n = 39) of the females and 57% (n = 7) of the males of the Damvallei testing positive. For the Walenbos population 42% (n = 53) of the females and 64% (n = 11) of the males showed *Wolbachia *infection. There was no difference between the sexes in infection frequency for both populations (Fisher's exact test: *P *> 0.2) and no difference in infection frequency between populations (*P *= 0.9). Prevalence of *Rickettsia *and *Cardinium *was fixed in the two investigated populations.

### (iii) Relationship between endosymbiont infection and sex ratio

**Figure 2:**
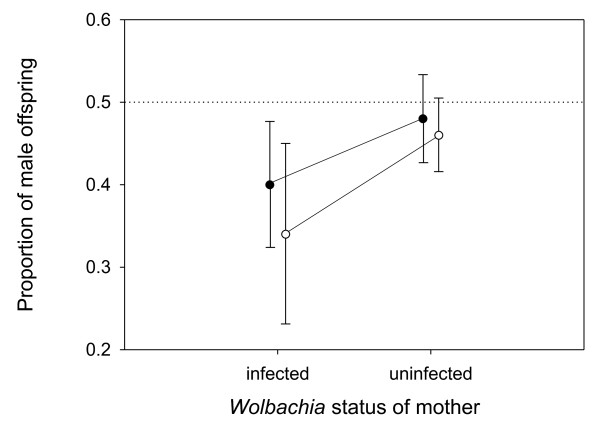
**Relationship between average proportion of male offspring in a clutch and *Wolbachia *infection status of the mother that produced the clutch in two different populations**. (filled circles = population Damvallei, unfilled circles = population Walenbos). Error bars indicate 95% confidence intervals. The dotted line depicts a 50:50 sex ratio.

As virtually no variation in infection frequency was observed for *Cardinium *and *Rickettsia*, female infection with these endosymbionts is unlikely to explain the maternal sex ratio variation. However, for *Wolbachia*, infection status of the female had a significant effect on tertiary sex ratio (*F*_1, 36.2 _= 6.61; *P *= 0.014; Figure 2), wherein infected females produced a significantly distorted sex ratio (mean ± SE: 0.36 ± 0.04; *t*_37 _= -3.70; *P *= 0.0007) while uninfected females produced a sex ratio that was not significantly different from 0.5 (mean ± SE: 0.47 ± 0.02; *t*_18.1_= -1.81; *P *= 0.086). The population a female originated from did not have a significant effect on the sex ratio (*F*_1, 36.2 _= 0.93; *P *= 0.340) and the effect of the *Wolbachia *infection was not different between both populations (*F*_1, 36.2 _= 0.20; *P *= 0.65). Within infection groups, no significant variation among females could be detected (estimated variance component: 0.008 ± 0.037; *P *= 0.59)

### (iv) Antibiotics treatment

**Figure 3:**
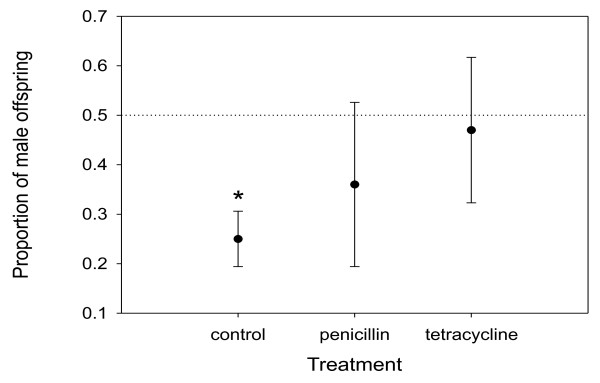
**Relationship between average proportion of male offspring in a clutch and antibiotics treatment applied to the mother**. Error bars indicate 95% confidence intervals. The dotted line depicts a 50:50 sex ratio. An asterisk indicates proportions significantly different from 0.5.

Sex ratio produced by females differed significantly among treatments (*F*_2, 34.6 _= 4.67; *P *= 0.016; Figure 3). While females of the control group produced a significantly distorted sex ratio (mean ± SE: 0.25 ± 0.03; t_37.7 _= -7.27; *P *< 0.0001), tetracycline treated females produced an even amount of males and females (mean ± SE: 0.47 ± 0.08; *t*_28.9 _= -0.38; *P *= 0.7). Although females treated with penicillin also produced a more even sex ratio compared to the control treatment (mean ± SE: 0.36 ± 0.08; *t*_40.2 _= -1.56; *P *= 0.126), there was no significant difference compared to the sex ratio of both control and the tetracycline treated females (Tukey post-hoc comparison: *t*_39.9 _= -1.35; *P *= 0.38 and *t*_35 _*= *-0.96; *P = *0.60 respectively). Although a single maternal line was used for the experiment, there was still significant variation among females (estimated variance component 0.55 ± 0.19; *P *= 0.002).

Antibiotic treatment significantly influenced the number of hatched offspring of the first clutch (*F*_2, 39 _= 5.14; *P *= 0.010). There was no significant difference in number of hatched offspring between control spiders (mean ± SE: 16.8 ± 1.6) and penicillin treated spiders (mean ± SE: 9.8 ± 2.2; Tukey post-hoc comparison: *t*_39 _= 2.25; *P *= 0.075). However a higher number of offspring from females treated with tetracycline (mean ± SE: 24.6 ± 4.5) hatched compared to the penicillin treatment (Tukey post-hoc comparison: *t*_39 _= -3.21; *P *= 0.007) but not compared to the control treatment (Tukey post-hoc comparison: *t*_33.9 _= -1.85; *P *= 0.167).

## Discussion

Results of our three independent experiments are consistent with the hypothesis that sex ratio variation in the solitary dwarf spider *Oedothorax gibbosus *is caused by reproductive manipulations by endosymbiont bacteria. First, as these bacteria are exclusively transmitted from mother to offspring by means of her eggs, daughters of sex ratio distorted females are expected to produce female distorted clutches as well. In line with this, our pedigree analysis shows that up to 70% of the sex ratio variation among females that is not attributed to sampling error is maternally inherited. Second, PCR-assays showed that *Oedothorax gibbosus *is infected with at least three different endosymbionts known to potentially affect reproductive behavior in arthropods namely: *Wolbachia, Rickettsia *and *Cardinium*. Out of these, *Wolbachia *certainly affects sex ratio in our study species; infected females produced a significantly female biased sex ratio compared to the even sex ratio of *Wolbachia *uninfected females. Third, the sex ratio manipulation by endosymbiont bacteria is further confirmed by treating distorted females of bacteria with the antibiotic tetracycline, which restored the production of equal amounts of males and females. Since juvenile survival was in general sufficiently high (>85%) and all female clutches were found for clutches with 100% juvenile survival, it is unlikely that differential juvenile mortality rates of males and females can account for the observed sex ratio bias. The combination of these results unequivocally demonstrates that, among other potentially distorting endosymbionts, *Wolbachia *is able to manipulate sex ratio in spiders. This represents to our knowledge the first clear evidence of a causal relationship between endosymbiont infection and its manipulative effect on host reproduction in spiders.

Several studies confirm the prevalence of several endosymbiont bacteria in the Araneae order, but convincing evidence of their effects on the hosts' reproductive biology is currently lacking. As argued by Weeks et al. [22], the establishment of multiple lines of evidence are a prerequisite to confirm the manipulative effect exhibited by these microorganisms. Previously, one such connection has been suggested by Gunnarsson et al. [21] who found an effect of female size on female post-copulatory position, which in turn affects brood sex ratio in *Pityohyphantes phrygianus*. This effect is altered by an interaction of the *Wolbachia *infection status of the male and infected female size which makes the observed effect indirect and subject to female control. Duron et al. [6] suggested manipulative effects by reporting a sex biased prevalence of *Wolbachia *in the spiders *Tetragnatha montana *and *Meta mengei*. It remains however unknown whether sex ratio distortion is present in those species.

Identifying the phylogenetic position of the *Wolbachia *strain of *Oedothorax gibbosus *might help to further explore the incidence of reproductive manipulations by *Wolbachia *in spiders. Based on the *wsp *sequences, the strain present in our study species is situated within supergroup G, previously reported by Rowley et al. [19] to be spider specific and has highest similarities with the strains found in the spider genera *Diaea *and *Dysdera*. These genera are known to exhibit primary sex ratio distortion (*Diaea *[15]) and parthenogenesis induction (*Dysdera *[12]). The classification of these sequences into a monophyletic distinct clade G is however not well supported, not only as it is merely based on a single gene, but additionally because it has been suggested to represent a recombinant of the supergroup A and B *wsp *sequences [25]. For 16S, unambiguous classification into one of the existing supergroups could not be supported, but it is most closely related to the sequences found in the spider *Tetragnatha montana*. Remarkably, this was also one of the two species out of the 26 spiders species tested by Duron et al. [6] where a manipulative effect of *Wolbachia *was suggested based on a higher prevalence in females compared to males.

Notwithstanding, whilst *Wolbachia *obviously plays an important role in the reproductive biology of *O. gibbosus*, our findings also point out that there is no simple one-to-one relationship between infection status and sex ratio distortion. First, if *Wolbachia *infection status alone was responsible for sex ratio variation, all among female variation would be expected to be inherited maternally. Our pedigree analysis in contrast revealed that a smaller though significant part of the among female variation is not strictly inherited from mother to daughters. Although the most straightforward explanation would be an imperfect transmission of the bacterium towards offspring, PCR screening demonstrated for a subset of the data that infected females (n = 4) only produced infected offspring (n = 47), indicating very high transmission efficiencies. Paternal effects, whether caused by meiotic drive, i.e. the unequal production of male and female producing sperm [27], or suppression of the manipulative effect by nuclear genes [28,29] are therefore more plausible mechanisms. Second, paternal effects are also suggested from the antibiotics treatment experiment; although a single maternal line was used there is still significant variation among females within the control treatment, which could be explained by the fact that females were mated with different males. Variation in *Wolbachia *titer, potentially induced by *Wolbachia *specific bacteriophages, may also substantially affect the expression of the manipulative effect [30]. It remains however at present less understood which factors determine *Wolbachia *concentrations in arthropods [31]. Third, a relative large proportion of males are still infected with *Wolbachia*, indicating an imperfect manipulative effect or the presence of resistance genes in both populations. *Wolbachia *infection of field captured individuals even suggests a higher prevalence in males compared to females, although this can be most probably attributed to sampling error. Indeed, given that the observed prevalence averaged over both populations equals 0.45, and that the observed sex ratio of infected females averages 0.36, the estimated proportion of individuals that are male and infected (i.e. males that originate from infected females) equals (0.45)*(0.36) = 0.162. Likewise, the proportion of individuals that are male and uninfected (i.e. males that originate from uninfected females) equals (0.55)*(0.5) = 0.275. Hence, the estimated proportion of infected males equals 0.162/(0.162 + 0.275) = 0.37. An exact binomial test however reveals that the observation of 11 infected males out of 18 males tested is not sufficient to reject the hypothesis that only 37% of the males are infected (*P *= 0.07).

Multiple species of endosymbionts were found to infect the same individual, which creates the opportunity for interaction effects to occur. Our use of several antibiotics that target different endosymbiont species allows to investigate the potential occurrence of such interaction effects. However, penicillin treated females, expected to only target *Cardinium*, did not result in a significantly different offspring sex ratio compared to the control and tetracycline treatment. Therefore no indications of the potential additional effect of *Cardinium *on the sex ratio could be observed.

It is known that *Wolbachia *can induce several reproductive alterations like parthenogenesis induction, male-killing and feminization [2,4,5]. Parthenogenesis induction is highly unlikely to occur since no offspring were produced by unmated females (pers. obs.). A female biased sex ratio can further be caused both by male killing and feminization. If male killing is present, the number of hatched offspring from infected females is typically half of that from uninfected females since male embryos are selectively killed. Feminization, however, converts genetic males into phenotypic females which results in an equal number of hatching offspring.

Based on our pedigree data, we found a significant positive correlation between clutch size and proportion of sons in each clutch, wherein smaller clutches were significantly more female biased. This points into the direction of killing of male embryos as the most likely manipulating mechanism. Nevertheless, more exclusive evidence could have been obtained from the antibiotics treatment. Although the higher hatching rate of tetracycline treated individuals approached twice the clutch size of control females, which is congruent with the expectations under male killing, this difference was not significant. The difficulty to clearly infer about the possible manipulating mechanism in our study species is most likely attributed to the large variance in clutch size (see Figure 1). Relationships between clutch size and sex ratio could therefore only be observed for the extensive pedigree data set.

Recognizing the effect of microorganisms on the reproductive biology of spiders is of high importance to further understand the mechanisms that cause the pronounced diversity of reproductive phenotypes observed in this arthropod order. At least for our male dimorphic study species [23], the sex ratio distorting effect of *Wolbachia *could be involved in the stable coexistence of both distinct male phenotypes through its influence on sexual selection, as suggested by theoretical [32] as well as empirical [33] investigations. In addition, our findings of an important role of endosymbionts on sex ratio distortion might provide a useful framework to decipher the mechanisms that cause parthenogenesis in spiders [14] and the facilitation of adaptive sex ratio adjustments that are commonly observed in social spiders to reduce male mate competition [9,34].

## Conclusions

In this study we adopted a threefold strategy to corroborate the role of endosymbiont bacteria in causing a female distorted sex ratio in the solitary dwarf spider *Oedothorax gibbosus*. (i) Pedigree analysis confirms that the sex ratio trait is primarily maternally inherited, (ii) PCR-assays show that individuals are infected with up to three endosymbionts known to cause reproductive alterations in arthropods, i.e. *Wolbachia*, *Rickettsia *and *Cardinium *and that females infected with *Wolbachia *produce significantly more females than males compared to uninfected females. (iii) Antibiotic treatment of *Wolbachia *infected females restores the production of an equal amount of males and females. This is the first direct evidence of endosymbiont interference in reproductive characteristics in the Araneae order. These findings can have major implications on understanding the mechanism causing the variety of reproductive phenotypes present in spider species.

## Methods

### (i) Maternal inheritance of sex ratio variation

To estimate the among female variance in clutch sex ratio and decompose it into a maternally inherited and residual component, a total of 192 females were captured in a wet forest ("Walenbos", Belgium) and mated with males from the same population. Offspring were bred for five consecutive generations in the lab, which resulted in a total of 3884 offspring originating from 414 different females. Spiders were reared individually in plastic vials of 5 cm diameter and 2,5 cm height. Plaster was added to the bottom and moistened to keep humidity levels at 100%. A piece of moss was provided to allow the construction of a functional web. The vials were placed in a climate chamber with a constant temperature of 20°C and light-dark regime of 16L-8D. Juveniles were fed with an overabundance of springtails (*Sinella curviseta*) and after the third moult an excess of fruit flies (*Drosophila sp.*) was provided. Vials were checked several times a week for food and humidity level. After reaching adulthood one male was placed in the vial of the female. The male was removed after at least 24h. Mated females were allowed to lay cocoons in the vial they were reared in. Sex was determined upon reaching adulthood by visual inspection using a stereomicroscope and tertiary sex ratio, defined as number of male offspring divided by the total number of adult offspring, was assessed.

Presence of a maternally inherited sex ratio variation and an estimate of its variance ($\sigma_{m}^{2}$) over multiple generations was obtained by means of an animal model [35] in which maternal effects were updated using Gibbs sampling. It is an extension of a model developed by [36] to estimate additive genetic effects for continuous traits in pedigrees.

More specifically, let *Y*_*i *_be the sex of offspring *i *and let *d,i *refer to the mother of the *i*'th offspring, then *Y*_*i *_was modelled following a Bernoulli distribution with mean *π*_*d,i*_, i.e. the sex ratio of the dam *d*, where

$$\ln\left(\frac{\pi_{d,i}}{1-\pi_{d,i}}\right)=\mu +m_{d,i}+e_{d,i}$$

with *μ *the average logit sex ratio in the population, *m*_*d,i *_the maternally inherited sex ratio effect and *e*_*d,i *_the sex ratio effect that is not captured in *m*_*d,i*_. Possible causes of *e*_*d,i *_to deviate from zero include additional variation in sex ratio among dams that is not attributed to sampling error. As sex ratio data are obtained over multiple generations in the pedigree, *m*_*d,i *_can be updated separately from *e*_*d,i *_by means of a recursive equation that sets the maternally inherited sex ratio effect of a particular mother equal to that of her daughters, i.o.w. *m*_*i *_= *m*_*d,i*_. Hence, *m*_*d,i *_of a particular dam is updated from both the sex ratio she produces as well as the sex ratio that her (grand)mother(s) and (grand)daughters produce. Following this procedure, the posterior distribution of the variance in *m*_*d,i *_and *e*_*d,i*_, i.e. $\sigma_{m}^{2}$ and $\sigma_{e}^{2}$ respectively, was obtained and used to calculate a point estimate based on the mean of the distribution and a 95% credibility interval (CI). The model was fitted using a Bayesian approach as implemented in the program WinBugs v.1.4. A gamma (0.1, 0.1) was chosen as a prior distribution for σ_*m *_and σ_*e*_. Two independent MCMC chains, each with different starting values, were run simultaneously for 12.000 generations. The first 2.000 generations were discarded as burn-in period.

We also fitted a more complex model that included a genetic component that contributes to the sex ratio variation. However, visual inspection of the independent MCMC's indicated that no convergence and, hence, no reliable estimates of the variance components could be obtained.

To explore which manipulation is involved, we correlated clutch size and clutch sex ratio. Ideally, killing of male offspring by endosymbiont bacteria reduces the clutch size to half the clutch size produced by non-manipulated females. Feminization and parthenogenesis on the other hand are expected to have no effect on clutch size. We related clutch size and clutch sex ratio by means of a Pearson correlation, wherein the estimate of the clutch sex ratio was weighed by the clutch size in order to down weight the inaccurate sex ratio estimates of small clutches.

### (ii) Endosymbiont detection and prevalence

Infection status and screening of endosymbionts was performed by means of PCR with endosymbiont specific primers. DNA was extracted from whole spiders with the Nucleospin Tissue kit (©Machery Nagel) following the manufacturers recommended protocol.

Spiders were screened for four different endosymbionts that are known to alter host reproduction in arthropods and that are already detected in spiders by means of the following specific primers: (i) WSP81F and WSP691R [7] to amplify a part of the cell surface protein coding gene of *Wolbachia *(*wsp*) and 16Swolb99F and 16Swolb994R [37] to selectively amplify the 16S ribosomal RNA gene of *Wolbachia*; (ii) CLO-f1 and CLO-r1 [38] to selectively amplify ~468bp part of the 16S rRNA gene of *Cardinium*; (iii) RICS741F and RICT1197R, which amplify a part of the *citrate *gene of *Rickettsia *[39] and (iv) SP-ITS-J04 and SP-ITS-N55 [40] to selectively amplify the spacer region between 16S and 23S rRNA genes of *Spiroplasma ixodetis*. To investigate whether *Wolbachia *detection in our study species was not confounded by the presence of *Wolbachia *in prey items, one sample of three fruitflies and one of 20 springtails were screened. None of these samples showed any traces of *Wolbachia*. PCR conditions were as follows: initial denaturation at 95°C for 2 min, followed by 35 cycles of denaturation at 94°C for 30 sec, annealing (54°C, 30 sec), extension (72°C, 90 sec) and a final extension at 72°C during 5 min. Electrophoresis was performed on a 1,5% agarose gel. The primers 3F and 9R [41], which amplify part of the 18S rDNA, were used as a positive control. Gels were stained in a solution of GELRED and bands were visualized by UV-fluorescence.

PCR products were sequenced using BigDye Terminator Sequencing mix and run on an ABI 3710 automated sequencer for a random sample of five to ten independent individuals to validate primer specificity and to test for the presence of different strains. Sequences were aligned with the ClustalW algorithm implemented in MEGA4 [42] with sequences from other spiders available in GenBank (mainly reported in the studies [6,17,20]). The closest relatives of the obtained endosymbiont sequences were identified by BLAST searches. Phylogenetic position and similarity of the endosymbionts with those of other spider species were analyzed based on a Bayesian Inference using MrBayes [43] with posterior probability support values calculated for the nodes. We assumed a general time reversible model of DNA substitution allowing a proportion of invariant sites and gamma distributed variation in substitution rate among sites (GTR + I + G) for all four DNA fragments. Four simultaneous chains (two cold, two heated) were run for two million generations and trees were sampled every 1000 generations. To check convergence and stability of the parameter estimates and to determine the burn-in value, we used Tracer 1.3 [44] to inspect the log files.

Prevalence of the bacteria was tested in two populations in Belgium, i.e. "Walenbos", being the same locality where spiders of the breeding experiment (i) originated from (n = 53 and n = 11 females and males respectively) and a second population "Damvallei" situated approximately 100 km westward (n = 39 and n = 7 females and males respectively).

### (iii) Relationship between endosymbiont infection and sex ratio

Spiders used for investigating the prevalence of the infections and testing the relationship between endosymbiont presence and sex ratio originated from hand catches carried out in the two populations mentioned earlier. Only juvenile and subadult spiders were used to ensure that females were virgin at the start of the experiment. Spiders were reared under the same conditions as mentioned above. At adulthood, females (n = 15 and n = 19 for Damvallei and Walenbos respectively) were mated with males of the same population and their offspring (n = 446 and n = 485 for Damvallei and Walenbos respectively) were reared individually till adulthood to determine offspring sex ratio and survival. Females were stored in ethanol after the production of three cocoons and afterwards screened for the presence of endosymbionts. If a female died before being stored, daughters were screened for endosymbiont presence. Only a single daughter was inspected if the result was positive and the mother was considered to be infected. In the case of a negative result, two additional daughters were screened and if these results were also negative the mother was considered to be uninfected. Offspring of a few females (number of females: n = 2 and n = 5 for Damvallei and Walenbos respectively) were reared for a second generation to increase sample size (number of offspring: n = 74 and n = 93 for Damvallei and Walenbos respectively). Significance of a relationship between endosymbiont infection and sex ratio was tested by means of a generalized linear mixed model (Proc GLIMMIX in SAS v.9.1.2) with endosymbiont presence/absence, population and their interaction as fixed effects. To correct for sex ratio variation among females which may inflate the error degrees of freedom, female was included as a random effect.

### (iv) Antibiotics treatment

To test whether an equal sex ratio can be restored after removal of the endosymbionts, we treated one highly distorted maternal line known to be infected with *Wolbachia*, *Rickettsia *and *Cardinium *with antibiotics. This line originated from the breeding experiment to test the relationship between endosymbiont infection and sex ratio distortion (see Methods section iii) and was kept in the lab for 7 generations. At generation 4, two females were mated with an unrelated male and 18 offspring from each female were randomly assigned to three different treatments i.e. (i) untreated (to continue the control line and reared as mentioned above), (ii) tetracycline hydrochloride treatment which eliminates *Wolbachia*, *Rickettsia *and *Cardinium *(0.1%, w/v; 0.002 M), (iii) penicillin treatment which only eliminates *Cardinium *(0.1%, w/v; 0.003 M)[38,45]. Antibiotics were applied by moistening the vials permanently with the antibiotics solution. Although tetracycline is bacteriostatic [46] and does not remove the bacteria but renders them inactive, PCR screening of antibiotics treated individuals demonstrated that tetracycline removed *Wolbachia *for about half of the specimens. For the remaining individuals, only faint PCR bands, often out of the expected range of the PCR product size were observed. After reaching adulthood, females of each treatment were mated with unrelated males and sex ratio and survival of the clutches was determined (number of females: n = 39, n = 7 and n = 8 for control, penicillin and tetracycline treatment respectively). Differences in average clutch sex ratio between the treatments were analyzed by means of a generalized linear mixed model (Proc GLIMMIX in SAS v. 9.1.2) with treatment as fixed effect. To account for dependence in sex ratio among clutches, mother (factor DAM) was included as a random effect. Survival was generally high and did not differ between spiders treated with tetracycline (mean ± SE: 0.86 ± 0.06), penicillin (mean ± SE: 0.87 ± 0.07) and control spiders (mean ± SE: 0.92 ± 0.02) (Generalized Linear Mixed Model; treatment effect: *F*_2, 40 _= 0.89; *P *= 0.42), indicating that administering antibiotics did not influence offspring survival.

## Authors' contributions

BV and FH conceived and designed the study and wrote the manuscript. BV, FH and JS analyzed the data and performed the breeding experiments and molecular analyses. All authors read and approved the manuscript.

## Supplementary Material

Additional file 1 **Phylogenetic position of *Wolbachia wsp *sequence of *Oedothorax gibbosus***. [GenBank:HQ286290]. Tree was constructed by Bayesian tree searching as implemented in MrBayes [43] on a subset of *Wolbachia *wsp sequences available at GenBank, with indication of the major *Wolbachia *supergroups. Node values represent posterior probabilities of the clades. Genbank accession numbers are given in front of the species name. Sequences that do not originate from spider hosts are preceded with the taxonomic group to which the host species belongs, spider hosts are underlined. *Oedothorax gibbosus *is shown in bold and italics. Click here for file

Additional file 2 **Phylogenetic position of *Wolbachia *16S rDNA sequence of *Oedothorax gibbosus***. [GenBank:HQ286291]. Tree was constructed by Bayesian tree searching as implemented in MrBayes [43] on a subset of *Wolbachia *16S rDNA sequences available at GenBank, with indication of the major *Wolbachia *supergroups. Node values represent posterior probabilities of the clades. Genbank accession numbers are given in front of the species name. Sequences that do not originate from spider hosts are preceded with the taxonomic group to which the host species belongs, spider hosts are underlined. *Oedothorax gibbosus *is shown in bold and italics. Click here for file

Additional file 3 **Phylogenetic position of *Rickettsia *(partial *citrate *sequence) endosymbiont of *Oedothorax gibbosus***. [GenBank:HQ286289]. Tree was constructed by Bayesian tree searching as implemented in MrBayes [43] on a subset of *Rickettsia *sequences available at GenBank. Node values represent posterior probabilities of the clades. Genbank accession numbers are given in front of the species name. Sequences that do not originate from spider hosts are preceded with the taxonomic group to which the host species belongs, spider hosts are underlined. *Oedothorax gibbosus *is shown in bold and italics. Click here for file

Additional file 4 **Phylogenetic position of *Cardinium *(16S rRNA gene) endosymbiont of *Oedothorax gibbosus***. [GenBank:HQ286292]. Tree was constructed by Bayesian tree searching as implemented in MrBayes [43] on a subset of *Cardinium *sequences available at GenBank. Node values represent posterior probabilities of the clades. Genbank accession numbers are given in front of the species name. Sequences that do not originate from spider hosts are preceded with the taxonomic group to which the host species belongs, spider hosts are underlined. *Oedothorax gibbosus *is shown in bold and italics. Click here for file
