# Supplementary figures and images for: Spiders do not escape reproductive manipulations by Wolbachia
Source: BMC Evol Biol. 2011 Jan 14;11:15. doi: 10.1186/1471-2148-11-15 (PMC3025852; doi:10.1186/1471-2148-11-15)

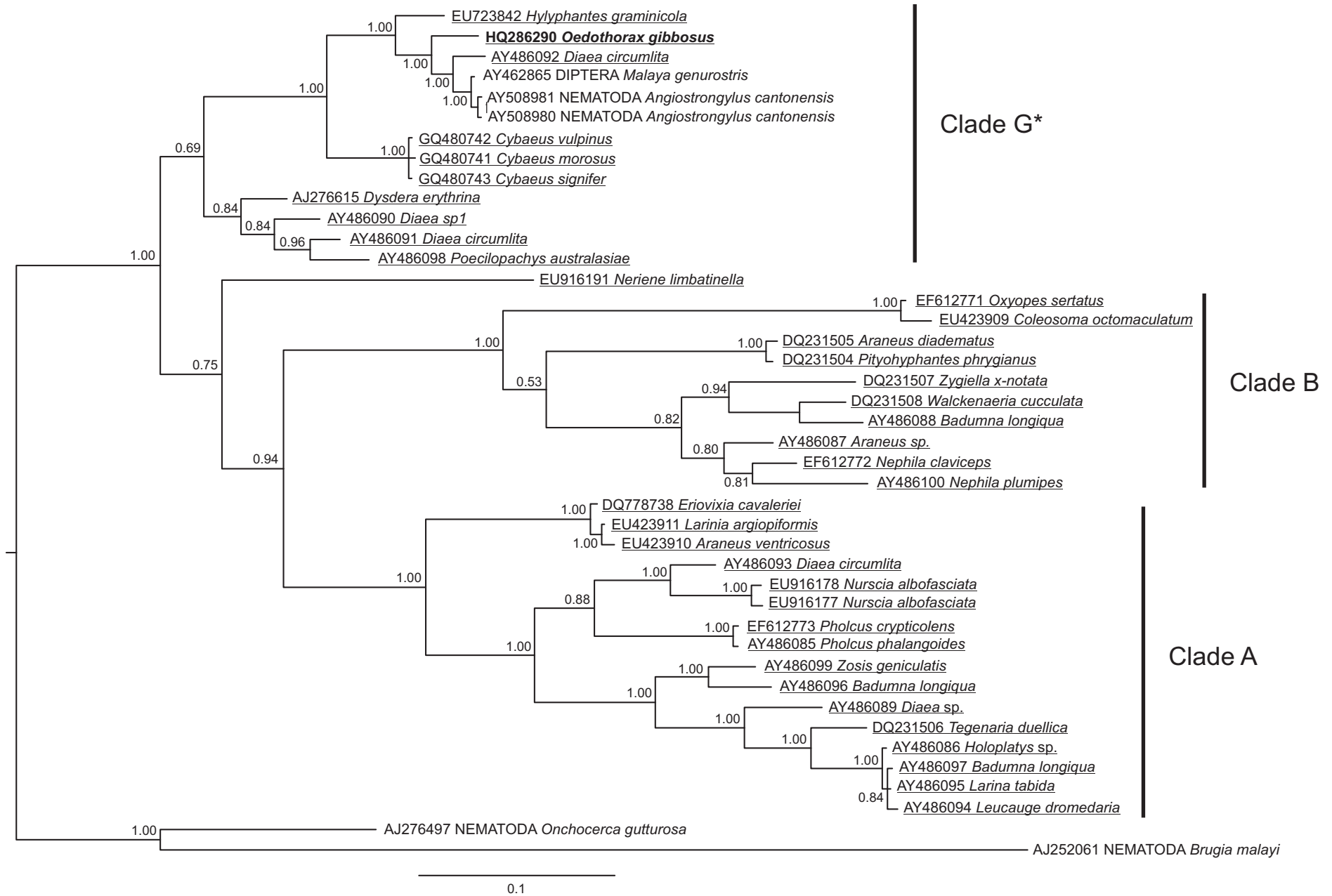

Supplement: Additional file 1 — Phylogenetic position of Wolbachia wsp sequence of Oedothorax gibbosus. [GenBank:HQ286290]. Tree was constructed by Bayesian tree searching as implemented in MrBayes [43] on a subset of Wolbachia wsp sequences available at GenBank, with indication of the major Wolbachia supergroups. Node values represent posterior probabilities of the clades. Genbank accession numbers are given in front of the species name. Sequences that do not originate from spider hosts are preceded with the taxonomic group to which the host species belongs, spider hosts are underlined. Oedothorax gibbosus is shown in bold and italics. [file 1471-2148-11-15-S1.PDF]

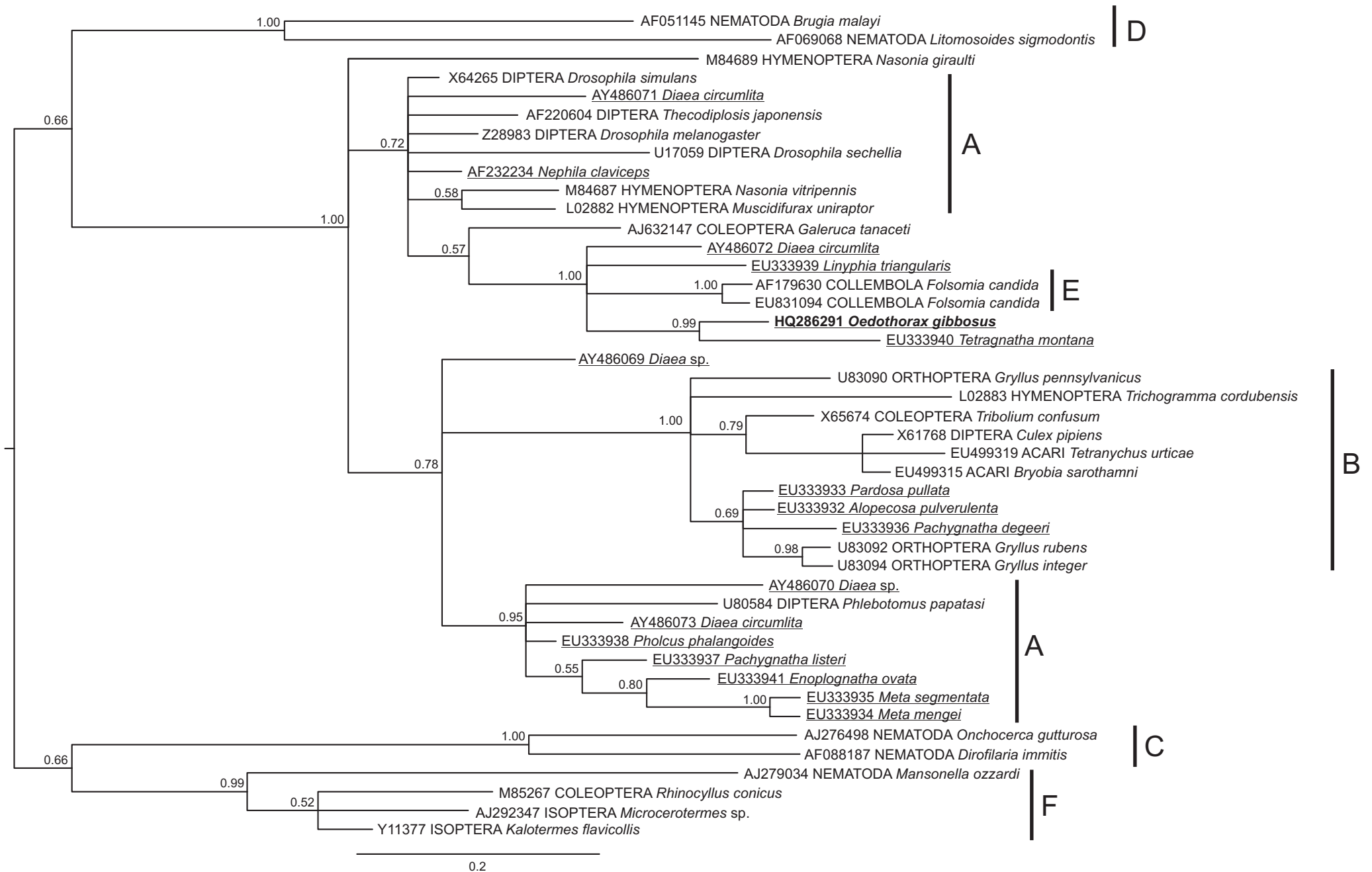

Supplement: Additional file 2 — Phylogenetic position of Wolbachia 16S rDNA sequence of Oedothorax gibbosus. [GenBank:HQ286291]. Tree was constructed by Bayesian tree searching as implemented in MrBayes [43] on a subset of Wolbachia 16S rDNA sequences available at GenBank, with indication of the major Wolbachia supergroups. Node values represent posterior probabilities of the clades. Genbank accession numbers are given in front of the species name. Sequences that do not originate from spider hosts are preceded with the taxonomic group to which the host species belongs, spider hosts are underlined. Oedothorax gibbosus is shown in bold and italics. [file 1471-2148-11-15-S2.PDF]

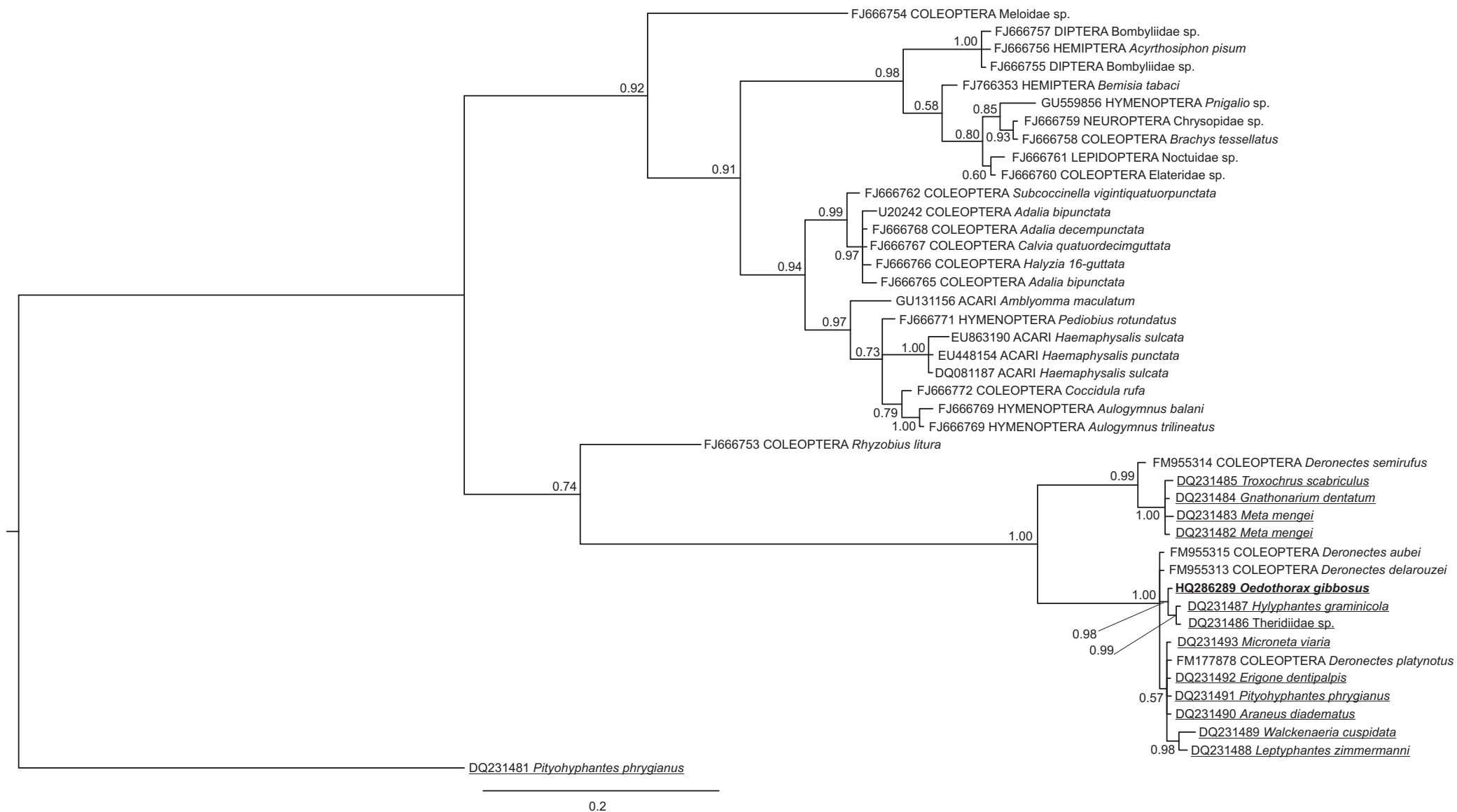

Supplement: Additional file 3 — Phylogenetic position of Rickettsia (partial citrate sequence) endosymbiont of Oedothorax gibbosus. [GenBank:HQ286289]. Tree was constructed by Bayesian tree searching as implemented in MrBayes [43] on a subset of Rickettsia sequences available at GenBank. Node values represent posterior probabilities of the clades. Genbank accession numbers are given in front of the species name. Sequences that do not originate from spider hosts are preceded with the taxonomic group to which the host species belongs, spider hosts are underlined. Oedothorax gibbosus is shown in bold and italics. [file 1471-2148-11-15-S3.PDF]

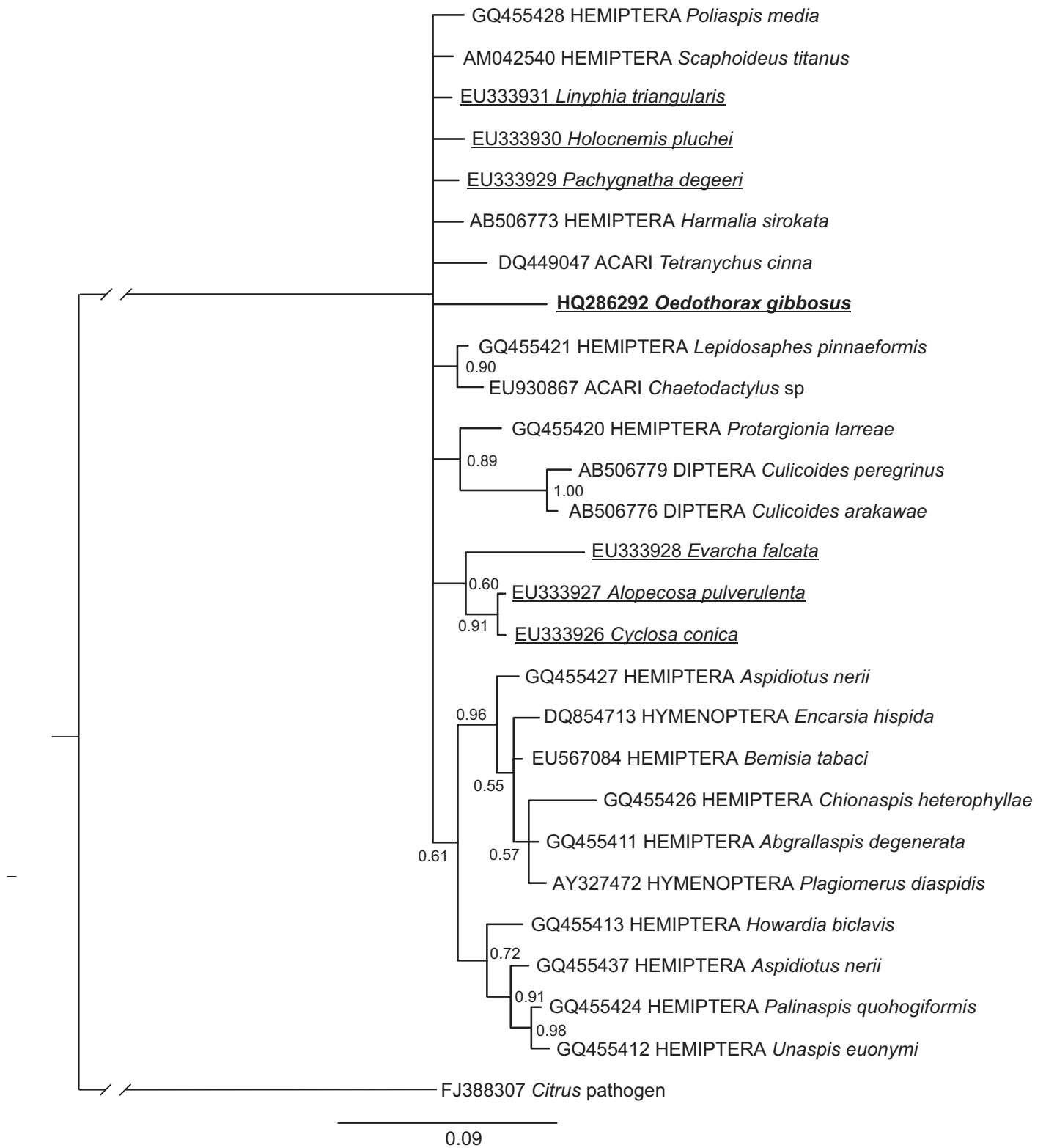

Supplement: Additional file 4 — Phylogenetic position of Cardinium (16S rRNA gene) endosymbiont of Oedothorax gibbosus. [GenBank:HQ286292]. Tree was constructed by Bayesian tree searching as implemented in MrBayes [43] on a subset of Cardinium sequences available at GenBank. Node values represent posterior probabilities of the clades. Genbank accession numbers are given in front of the species name. Sequences that do not originate from spider hosts are preceded with the taxonomic group to which the host species belongs, spider hosts are underlined. Oedothorax gibbosus is shown in bold and italics. [file 1471-2148-11-15-S4.PDF]
